# Complete genome analysis of a rare human G3P[9] rotavirus posing as an AU-1 like strain

**DOI:** 10.1186/2193-1801-2-569

**Published:** 2013-10-26

**Authors:** Apiradee Theamboonlers, Ornwalan Maiklang, Thanunrat Thongmee, Thaweesak Chieochansin, Viboonsuk Vuthitanachot, Yong Poovorawan

**Affiliations:** Center of Excellence in Clinical Virology, Department of Pediatrics, Faculty of Medicine, Chulalongkorn University and Hospital, Bangkok, 10330 Thailand; Chum Phae Hospital, Chum Phae, Khon Kaen, Thailand

**Keywords:** Human rotavirus (HRV), Uncommon strain, AU-1 like strain, G3P[9]

## Abstract

**Background:**

We performed phylogenetic and sequence analysis by Basic Local Alignment Search Tool (BLAST) of a complete Human Rotavirus (HRV) genome isolated from a hospitalized child with acute gastroenteritis in Thailand.

**Findings:**

The results indicated an uncommon strain characterized by multiple re-assortments in the VP3, VP4, VP6, NSP1, NSP4 and NSP5 genes. The uncommon strain is genotype G3-P[9]-I3-R3-C3-M3-A3-N3-T3-E3-H6, which displays aspects of the AU-1, FRV-1 and corresponds to the feline/canine prototype G3P[9] strain.

**Conclusions:**

The results suggested that nearly all the eleven gene segments of G3P[9] RVA strain CU365 might have originated from feline/canine RVAs (Rotavirus A).

## Findings

Rotavirus group A is the most common cause of severe acute gastroenteritis among infants and young children with a mortality rate of 454,000-705,000 annually in children under 5 years of age especially in developing countries (Parashar et al. [2006]). The predominant mode of transmission is by fecal-oral route and the dramatic spread occurs in winter. Rotavirus is a member of the *Rotavirus* genus, *Reoviridae* family and has a double stranded RNA genome. The viral capsid is triple-layered; the core layer contains the virus genome that is characterized by 11 segments encoding either structural viral proteins VP7, VP4, VP6, VP1, VP2 and VP3 or nonstructural NSP1, NSP2, NSP3, NSP4 and NSP5 genes which show the genotype constellation of Gx-P[x]-Ix-Rx-Cx-Mx-Ax-Nx-Tx-Ex-Hx, respectively (Matthijnssens et al. [2011a]). The outer capsid protein consists of VP7, glycoprotein or G-protein determines the type specificity of G and VP4, protease-cleaved or P- protein determined P types. The four common strains G1P [8], G3P [8], G4P [8] and G2P [4] of rotavirus predominate globally but the prevalence of strains can vary geographically and unusual strains also can occur. The G3 strains are the genotype with a broad host range and have been detected in several host species. Apart from its impact on human health, rotavirus also infects animals and is a pathogen of domestic animals. According to previous reports, uncommon rotavirus strains sharing genetic features of various virus strains have been isolated from humans and animals (Matthijnssens et al. [2011b]). Direct interspecies transmission and exchange of dsRNA segments via re-assortment may account for the detection of animal rotavirus strains in humans or vice versa (Palombo [2002]). The P[9] rotavirus strain’s frequency amounts to less than 2.5% worldwide, while it is common in felines (Khamrin et al. [2007]). Although the G3P[9] strain is rarely detected in humans it has been documented in Japan, Thailand and Spain (Khamrin et al. [2007]; Iizuka et al. [1994]; Sánchez-Fauquier et. al. [2006]; Kaga et al. [1994]).

The study was approved by the Ethics Committee, Faculty of Medicine, Chulalongkorn University, Thailand (IRB number 129/52). A 14-month-old boy was admitted to the district hospital, Chum Phae, Khon Kaen province, northeast Thailand in November 2008. He had a 2-day history of fever and watery diarrhea and was diagnosed with acute gastroenteritis. He received supportive treatment and intravenous fluid to compensate for dehydration. He was discharged after 2 days of admission with complete recovery. His stool (CU365) was sent to the Center of Excellence in Clinical Virology for etiological investigation, and it was found to be positive for rotavirus RNA by RT-PCR (reverse transcriptase polymerase chain reaction). Briefly, viral RNA was extracted by RBC Viral Nucleic Acid Extraction Kit ( RBC Bioscience, Taipei, Taiwan) following the manufacturer’s instructions. RT-PCR of the complete genome was performed using specific primer sets (available on request) under conditions described previously (Theamboonlers et al. [2005]). To purify the PCR products, the HiYield Gel/PCR DNA fragments extraction Kit (RBC Bioscience, Taipei, Taiwan) was used according to the manufacturer’s instructions. Complete genome sequencing using specific primers was performed by First BASE Laboratories Shd Bhd (Salangor Darul Ehsan Malaysia). For sequence analysis, BLAST analysis was performed subsequently. (http://blast.ncbi.nlm.nih.gov/Blast.cgi, http://rotac.regatools.be/). The strain sequences were submitted to the GenBank database under accession numbers: JN706577 for NSP1 gene, JN706599 for NSP2 gene, JN706621 for NSP3 gene, JN 706643 for NSP4 gene, JN706665 for NSP5 gene, JN 706445 for VP1 gene, JN706480 for VP2 gene, JN706511 for VP4 gene, JN706508 for VP3 gene, JN706533 for VP6 gene, JN706555 for VP7 gene of the studied strain.

**Figure 1:**
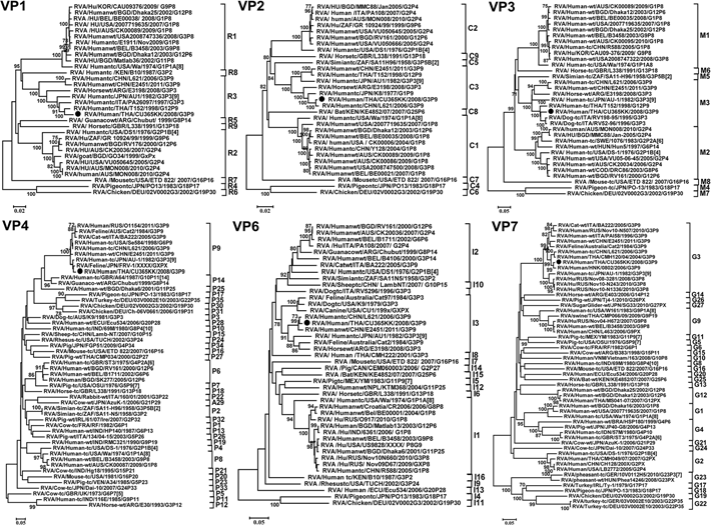
**Phylogenetic trees constructed from the nucleotides sequences of VP1, VP2, VP3, VP4, VP6 and VP7 genes from this current study with reference strains from GenBank.**

**Figure 2:**
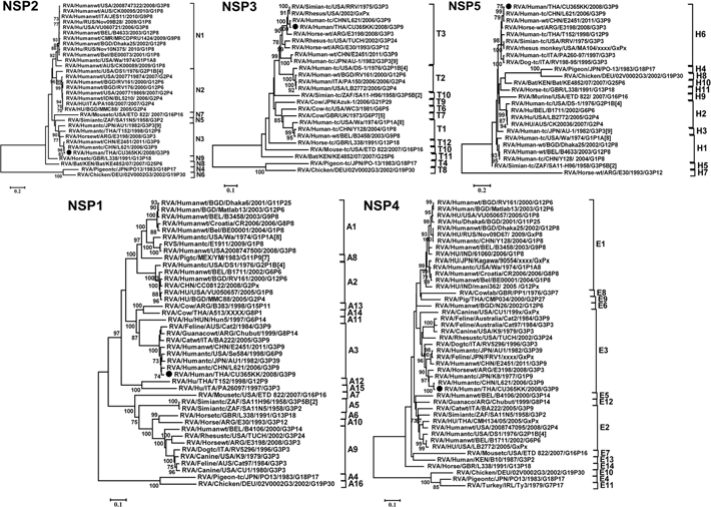
**Phylogenetic trees constructed from the nucleotides sequences of NSP1, NSP2, NSP3, NSP4 and NSP5 genes from this current study with reference strains from GenBank.**

Based on all eleven RNA segments, the uncommon strain (CU365) showed a genotype of G3-P[9]-I3-R3-C3-M3-A3-N3-T3-E3-H6 which suggested a close relationship with L621 human rotavirus A strain RVA/Human-tc/CHN/L621/2006/G3P[9] (Wang et al. [2013]) in NSP2, NSP3, NSP4, VP2, VP6 genes with 99% nucleotide identity by BLAST analysis which might have originated from feline/canine RVA strains including AU-1 like strains and the prototype strain BA222 of genotype G3-P[9]-I3-R3-C3-M3-A3-N3-T3-E3-H3 constellation (Matthijnssens et al. [2011a]; Iizuka et al. [1994]; Kaga et al. [1994]). In addition, the nucleotide identity between CU 365 and rotavirus AU-1 which is related to bovine rotaviruses (Nakagomi and Kaga [1995]) for the NSP1 gene amounted to 99% identity. Moreover, the nucleotide identity between CU365 and the RVA/Human-tc/THA/T152/1998/G12P[9] (Rahman et al. [2007]) strain’s VP1 gene was found to be 97%. Interestingly, the percent nucleotide identity between our study strain and the canine strain was also demonstrated to be 96% in VP3 (Matthijnssens et al. [2011b]). In addition, the nucleotide identity between the VP4 gene of CU365 and human rotavirus strain AU-1 (Isegawa et al. [1992]) was shown to be 99%. The nucleotide identity between the VP7 gene of CU365 and strain 0802 of the emerging G3 rotaviruses in Hong Kong detectable in a 3-year-old boy (Mitui et al. [2011]) was found to be 99%. The comparison between the observed genotype constellation for CU365 and other related known human and animal RVA genotype constellation is shown in Table 1. Multiple sequence alignments were compared using Clustal W v1.83 and phylogenetic trees were created with MEGA software v4.1 using the neighbor-joining method of measuring phylogenetic distances. A maximum likelihood tree derived from 1000 bootstraps was constructed to show the relationship between the recently re-assorted Thai HRV strain CU365 and other HRV strains obtained from GenBank (Figures 1 and 2).

**Table 1:** **Complete genotype constellations of known completely sequenced, CU365, DS-1, Wa, AU-1-like, FRVs, CRVs and feline/canine-like HRV strains**

Strain name	Genogroup	Genomic constellation										
		VP7	VP4	VP6	VP1	VP2	VP3	NSP1	NSP2	NSP3	NSP4	NSP5
RVA/Human-tc/jpn/AU-1/1982/G3P3[9]	AU-1-like	G3	P[9]	I3	R3	C3	M3	A3	N3	T3	E3	H3
RVA/Human-tc/CHN/L621/2006/G3P[9]	AU-1-like	G3	P[9]	I3	R3	C3	M3	A3	N3	T3	E3	H6
RVA/Human-wt/CHN/E2451/2011/G3P[9]	AU-1-like	G3	P[9]	I3	R3	C3	M3	A3	N3	T3	E3	H6
**RVA/Human-tc/THA/CU-365/2008/G3P[9]**	**AU-1-like**	**G3**	**P[9]**	**I3**	**R3**	**C3**	**M3**	**A3**	**N3**	**T3**	**E3**	**H6**
RVA/Human-tc/THA/t152/1998/G12P[9]	AU-1-like	G3	P[9]	I3	R3	C3	M3	A12	N3	T3	E3	H6
RVA/Human-tc/ITA/PA260-97/1997/G3P[3]	AU-1-like and Cat97-like	G3	P[3]	I3	R3	C3	M3	A15	N2	T3	E3	H6
RVA/Dog-tc/ISR/Ro1845/1985/G3P3	Cat97-like	G3	P[3]	I3	R3	C3	M3	A9	N2	T3	E3	H6
RVA/Dog-tc/ITA/RV198-95/1995/G3P3	Cat97-like	G3	P[3]	I3	R3	C3	M3	A9	N2	T3	E3	H6
RVA/Cat-tc/AUS/Cat2/1984/G3P[9]	Cat97-like and BA222-05-like	G3	P[3]	I3	R3	C3	M3	A3	N1	T6	E3	H3
RVA/Cat-wt/ITA/BA222/2005/G3P[9]	BA222-05-like	G3	P[9]	I2	R2	C2	M2	A3	N1	T3	E2	H3
RVA/Human-wt/ITA/PAH136/1996/G3P[9]	BA222-05-like	G3	P[9]	I2	R2	C2	M2	A3	N1	T6	E2	H3
RVA/Human-wt/ITA/PA158/1996/G3P[9]	BA222-05-like	G3	P[9]	I2	R2	C2	M2	A3	N2	T6	E2	H3
RVA/Human-DS-1	DS-1	G2	P[4]	I2	R2	C2	M2	A2	N2	T2	E2	H2
RVA/Human-Wa	Wa	G1	P[8]	I1	R1	C1	M1	A1	N1	T1	E1	H1

Strain in bold has been invesgated in this study.

It has been found in previous studies that genotype G3 can be detected in several host species including humans, rabbits, monkeys, pigs, birds, cats, dogs, horses, mice, cows and lamb (Estes and Kapikian [2007]; Martella et al. [2003]). The P[9] genotype has also frequently been isolated from humans, cats and dogs and hence, has been named the feline/canine-like strain (Wang et al. [2013]; Nakagomi et al. [1985]; Mochizuki et al. [1997]). A previous report on children afflicted with diarrhea in Japan showed that these children carried an AU-1-like and FRV-1-like G3P[9] rotavirus prototype strain (Iizuka et al. [1994]; Kaga et al. [1994]), which suggests an interspecies transmission event from cats/dogs to humans. The previously reported complete genome analysis investigated two rare G3P[9] rotavirus (PAH136/96 and PAI58/96) isolated from children with diarrhea which were recognized as assortments of genes closely related to rotaviruses from cats, ruminants and humans. The results suggested multiple transmissions of genes from animal to human strains of rotaviruses (De Grazia et al. [2010]) identical to the previous study of complex evolutionary patterns of two rare human G3P[9] rotavirus strains also possessing a feline/canine-like H6 genotype on an AU-1-like genotype constellation (Wang et al. [2013]). Other reports on similar reassortment include AU-1-like human G3P[9] strains detected in USA and Italy which also showed reassortments with canine and feline G3P[9] strains or other human strains. The full-length genome sequence of a feline G3P[9] rotavirus (RV) strain, BA222, identified from the intestinal content of an adult cat, was determined. Strain BA222 possessed a G3-P[9]-I2-R2-C2-M2-A3-N1-T3-E2-H3 genomic constellation, differing substantially from other feline RVs (De Grazia et al. [2010]; Grant et al. [2011]; De Grazia et al. [2008]; Martella et al. [2011]). In addition, three G3P[9] rotaviruses, detected in children hospitalized with gastroenteritis in Palermo, Italy, were found to be genetically related to strains of either human or feline origin in the VP7, VP4, and VP6 genes. In contrast, in the NSP4 gene the viruses resembled G2P[4] human strains, suggesting a reassortment between AU-1-like and Kun-like strains (De Grazia et al. [2008]). From these results suggested that future studies of cat and dog stool specimens would be of interest to survey the frequency of interspecies transmission, to monitor the co-evolution of human and animal rotavirus strains and to identify the human/animal reassorted viruses (Grant et al. [2011]). Altogether, the findings suggest that feline RVs are genetically diverse and that human RVs may occasionally originate either directly or indirectly (via reassortment) from feline RVs (Martella et al. [2011]). The new RV classification system, established by Matthijnssens et al. (Matthijnssens et al. [2011b]) allows data to accumulate rapidly in order to elucidate the genetic relationships among human and animal RV strains. Overall, the findings of previous studies have demonstrated that gathering information on animal viruses is fundamental to better understand the evolution of human RVs, especially those that do not belong to the common Wa like and DS-1-like families (Martella et al. [2011]).

In conclusion, we have described the recent RV re-assortment of the VP1, VP2, VP4, VP6, VP7, NSP1, NSP2, NSP3, NSP4 and NSP5 genes in a strain isolated from a child in Thailand in 2008 with characteristics consistent with strain 621 from China-Wuhan which is the AU-1-like and canine/feline-like RV strain. Interestingly, the VP3 gene in this study was closely related to the canine strain RV52-96. This is the first report on complete genome analysis of G3P[9] RVAs from Thailand, and our results indicate that feline/canine RVA can be transmitted to a human host. This finding highlights the risk of re-assorted RV transmission between humans and animals. In addition, due to the apparently random nature of RVA re-assortment, it underscores the need for future studies on molecular characterization of whole genomes of RVAs in order to detect potentially important viral re-assortments.

### Consent

Written informed consent was obtained from the patient’s guardian/parent/next of kin for the publication of this report and any accompanying images.
